# Design and analysis of modified octagonal ring-shaped MIMO antenna with connected ground for 5G sub-6 GHz n79, Wi-Fi 5, and Wi-Fi 6E band applications

**DOI:** 10.1038/s41598-026-58359-4

**Published:** 2026-06-27

**Authors:** Tathababu Addepalli, Sivasubramanyam Medasani, K Sathish, J Prasanth Kumar, M Balaji, Rajeev Kumar, Ashish Kumar, Dinesh Kumar Singh, Manish Sharma, Ashish Pandey

**Affiliations:** 1 Department of ECE, Aditya University, Surampalem, India; 2 Department of CSE, K. S. School of Engineering and Management, Bengaluru, India; 3 Department of ECE, Saveetha School of Engineering, Saveetha Institute of Medical and Technical Sciences (SIMATS), Chennai, Tamil Nadu 602105 India; 4 Department of ECE, Ramachandra College of Engineering, Eluru, Andhra Pradesh India; 5 Department of ECE, School of Engineering, Mohan Babu University, [Erstwhile Sree Vidyanikethan Engineering College], Tirupati, Andhra Pradesh India; 6 Chitkara University Institute of Engineering and Technology, Chitkara University, Chandigarh, Punjab India; 7 Electronics and Communication Engineering Department, GL Bajaj Institute of Technology and Management, Greater Noida, UP India; 8 Department of Electrical, Electronics and Communication Engineering, Galgotias University, Greater Noida, Uttar Pradesh 203201 India; 9 Department of Data Science and Engineering, Manipal University Jaipur, Jaipur, Rajasthan India

**Keywords:** Four-port MIMO, Octagonal ring, Connected-ground, 5G sub-6 GHz n79, Wi-Fi 5, Wi-Fi 6E, Mutual coupling reduction, Specific absorption rate (SAR), Engineering, Physics

## Abstract

The increasing demand for compact, high-isolation, and wideband antennas in modern wireless systems has created the need for efficient multiple-input multiple-output (MIMO) antenna designs suitable for 5G sub-6 GHz, Wi-Fi 5, and Wi-Fi 6E applications. MIMO antennas improve channel capacity, spectral efficiency, link reliability, and multipath performance, but maintaining compactness, wide bandwidth, low mutual coupling, and SAR compliance remains a major design challenge. This work proposes and experimentally validates a compact four-port modified octagonal ring-shaped MIMO antenna with a connected-ground structure for the 5G n79, Wi-Fi 5, and Wi-Fi 6E frequency bands. The antenna is designed on a low-cost FR4 substrate with dimensions of 60 × 60 mm². Four modified octagonal ring-shaped radiating elements are arranged orthogonally to improve polarization and spatial diversity. A connected-ground structure with multiple strips and slits is introduced to enhance impedance matching and reduce mutual coupling. The antenna is analyzed through design evolution, surface-current distribution, parametric optimization, S-parameters, far-field radiation characteristics, diversity parameters, and SAR evaluation using a human-head phantom model. The fabricated prototype is measured and compared with the simulated results. The proposed MIMO antenna achieves a wide impedance bandwidth of approximately 3.3–7.7 GHz, covering the 5G sub-6 GHz n79, Wi-Fi 5, and Wi-Fi 6E bands. Mutual coupling remains below − 17.5 dB across the operating band. The antenna exhibits stable radiation characteristics, with peak realized gain varying from about 4.3 dBi to 5.58 dBi and radiation efficiency between 80 and 96%. The measured and simulated results show good agreement. The diversity performance is satisfactory, with the envelope correlation coefficient close to zero, diversity gain nearly 10 dB, total active reflection coefficient below − 10 dB, channel capacity loss below the acceptable limit, and balanced MEG values. SAR values are also within the Federal Communications Commission (FCC) and International Commission on Non-Ionizing Radiation Protection (ICNIRP) safety limits for both 1 g and 10 g tissue models. The proposed connected-ground four-port MIMO antenna provides compact size, wide impedance bandwidth, high isolation, stable radiation performance, good diversity characteristics, and SAR compliance. Therefore, it is a promising candidate for 5G sub-6 GHz n79, Wi-Fi 5, Wi-Fi 6E, and other high-data-rate wireless communication systems.

## Introduction

The rapid growth of fifth-generation (5G) sub-6 GHz communication systems, Wi-Fi 5, Wi-Fi 6E, Internet of Things (IoT), and compact wireless terminals has increased the demand for antennas that can simultaneously provide wide impedance bandwidth, compact size, stable radiation characteristics, high radiation efficiency, low mutual coupling, and safe electromagnetic exposure levels. In practical wireless devices, multiple frequency bands are often required in a limited physical space; therefore, a single compact antenna system capable of covering several communication standards is preferred over multiple independent antennas. However, the integration of multiple radiating elements in a compact platform introduces design challenges such as impedance mismatch, high inter-element coupling, distorted radiation patterns, degraded diversity performance, and possible specific absorption rate (SAR) concerns when the antenna is placed close to the human body.

Multiple-input multiple-output (MIMO) antenna technology is widely adopted in modern wireless communication systems because it improves channel capacity, link reliability, spectral efficiency, and multipath fading performance without requiring additional spectrum or transmit power. In MIMO systems, low mutual coupling among antenna elements is essential to obtain good diversity performance. Several four-port MIMO antennas have been reported for sub-6 GHz, IoT, WLAN, and 5G applications. Compact wideband four-port MIMO antennas for sub-6 GHz and IoT applications have demonstrated the usefulness of orthogonal element placement and ground modification techniques for bandwidth enhancement and isolation improvement^1^. Different compact and high-gain four-port MIMO configurations have also been reported for 5G communication systems, including dual-band, n78 band, penta-band, UWB, and flexible wearable antenna structures^2–6^. These designs show good performance in their respective operating bands; however, many of them either focus on narrow or dual operating bands, require additional decoupling structures, or do not simultaneously address 5G n79, Wi-Fi 5, Wi-Fi 6E, and SAR-compliant operation in a compact connected-ground configuration.

Several researchers have explored wideband, super-wideband, and multiband MIMO antennas by using slotted patches, metasurfaces, machine-learning-assisted optimization, cross-polarized structures, characteristic mode analysis, and modified radiating elements^7–13^. Reconfigurable and quad-element MIMO antennas have also been proposed to improve frequency agility, isolation, and diversity performance for 5G communication systems^14–16^. Although these designs provide useful solutions for wideband and multiband operation, their implementation may involve complex geometries, additional isolation networks, parasitic structures, or larger antenna footprints. In addition, some reported designs mainly emphasize impedance bandwidth and isolation, while limited attention is given to the combined evaluation of measured S-parameters, diversity metrics, far-field radiation characteristics, and SAR performance.

Recent works published in Scientific Reports and other journals have further demonstrated the relevance of compact MIMO antennas for sub-6 GHz, mm-wave, satellite, IoT, V2V, and wearable applications^17–25^. High-isolation sub-6 GHz MIMO antennas, mm-wave MIMO structures, frequency-selective surface integrated antennas, and compact planar UWB antennas have shown promising results for advanced wireless systems. Other studies have focused on millimeter-wave, terahertz, automotive, and super-wideband MIMO antennas using different radiating geometries and isolation improvement methods^26–35^. However, many of these antennas are designed for either higher-frequency bands, ultra-wideband systems, or application-specific frequency ranges, and they may not directly provide a compact low-cost FR4-based solution for the combined 5G sub-6 GHz n79, Wi-Fi 5, and Wi-Fi 6E bands. Furthermore, the issue of common connected ground is particularly important for practical integration because fully separated ground planes may improve isolation in simulation but may not represent a realistic common-reference ground environment in compact wireless devices.

Another important concern for antennas intended for handheld and portable wireless devices is SAR compliance. Antennas operating near the human body must satisfy regulatory exposure limits defined for 1 g and 10 g tissue models. Several studies have investigated SAR behavior and maximum permissible input power for compact antennas and mobile communication systems^36,37^. The importance of practical design considerations in printed MIMO antenna systems, including ground configuration and realistic integration issues, has also been highlighted in the literature^38^. Tissue dielectric properties required for SAR estimation are commonly obtained from standard databases and are used for evaluating antenna safety near biological tissues^39^. Therefore, a compact MIMO antenna design that combines wideband operation, connected-ground practicality, high isolation, stable radiation performance, good diversity characteristics, and SAR compliance is highly desirable^40^. A four-port MIMO antenna for 5G New Radio has been optimized using machine-learning algorithms, with K-nearest neighbor (KNN) reported as the most suitable method. A dual-band antenna operating at 24.0 GHz and 32.0 GHz utilizes MIMO-RDRA and achieves isolation greater than 27.0 dB^41,42^. Cognitive-radio/DRA integrated concepts have also been reported^43–45^ and operate in all-pass filter, interweave, and underlay modes. A two-port sickle-shaped MIMO antenna sharing a common ground produces two narrow bands, 2.40–2.80 GHz and 4.48–5.75 GHz^46,47^. A two-port MIMO antenna has been validated by equivalent-circuit model analysis^48–50^, where the RLC components are calculated for the antenna resonances. Machine-learning algorithms have also been used for antenna optimization^51^, where S11 is predicted using K-nearest neighbor (KNN), extreme gradient boosting (XGBoost), decision tree (DT), and random forest (RF) methods.

In this work, a compact four-port modified octagonal ring-shaped MIMO antenna with a connected-ground structure is proposed for 5G sub-6 GHz n79, Wi-Fi 5, and Wi-Fi 6E band applications. The antenna is fabricated on a low-cost FR4 substrate with an overall size of 60 × 60 mm². Four identical modified octagonal ring-shaped radiating elements are arranged orthogonally to improve spatial and polarization diversity. Unlike several reported structures that use isolated ground planes or additional decoupling networks, the proposed design employs a connected-ground configuration with multiple strips and slits to provide a practical common-reference ground while maintaining wide impedance bandwidth and low mutual coupling. The design evolution, surface-current distribution, and parametric analysis are presented to explain the role of the modified radiator and connected-ground structure. The fabricated prototype is experimentally validated in terms of reflection coefficient, transmission coefficient, peak realized gain, radiation efficiency, and 2D radiation patterns. In addition, key MIMO diversity parameters such as envelope correlation coefficient, diversity gain, total active reflection coefficient, mean effective gain, and channel capacity loss are evaluated. SAR analysis is also performed using a human-head phantom model for both 1 g and 10 g tissue standards to confirm compliance with FCC and ICNIRP limits.

The main novelty and contributions of this work are as follows: first, a modified octagonal ring-shaped four-port MIMO antenna is developed to cover the 5G n79, Wi-Fi 5, and Wi-Fi 6E bands within a compact 60 × 60 mm² footprint. Second, a connected-ground structure with optimized strips and slits is introduced to achieve a practical common-ground configuration while maintaining mutual coupling below − 17.5 dB across the operating band. Third, the proposed design provides a wide impedance bandwidth of approximately 3.3–7.7 GHz, a stable gain of about 4.3–5.58 dBi, and radiation efficiency of 80%–96%. Fourth, the antenna demonstrates good MIMO diversity performance with ECC close to zero, DG close to 10 dB, TARC below − 10 dB, and acceptable CCL and MEG values. Finally, the proposed antenna is validated through simulation, fabrication, measurement, and SAR analysis, confirming its suitability for compact 5G and WLAN-enabled wireless devices.

The specific design objectives are summarized as follows:

- The single-port antenna is designed on an FR4 substrate, generating a − 10 dB impedance bandwidth of 3.20–8.27 GHz with overall dimensions of 25 × 38 mm²;
- The four-port MIMO antenna configuration is formed by placing four single radiating patches in an orthogonal arrangement;
- The size of the MIMO antenna is 60 × 60 mm²;
- The addition of asymmetric ground slits improves both isolation and bandwidth;
- The evolution of the four-port MIMO antenna is validated using surface-current-density analysis, and the relevance of the ground slits is explained;
- The SAR performance is within the acceptable limits for 1 g and 10 g tissue models;
- The maximum peak realized gain is 5.58 dBi in the operating band.

## Single-port antenna design and evolution

**Fig. 1:**
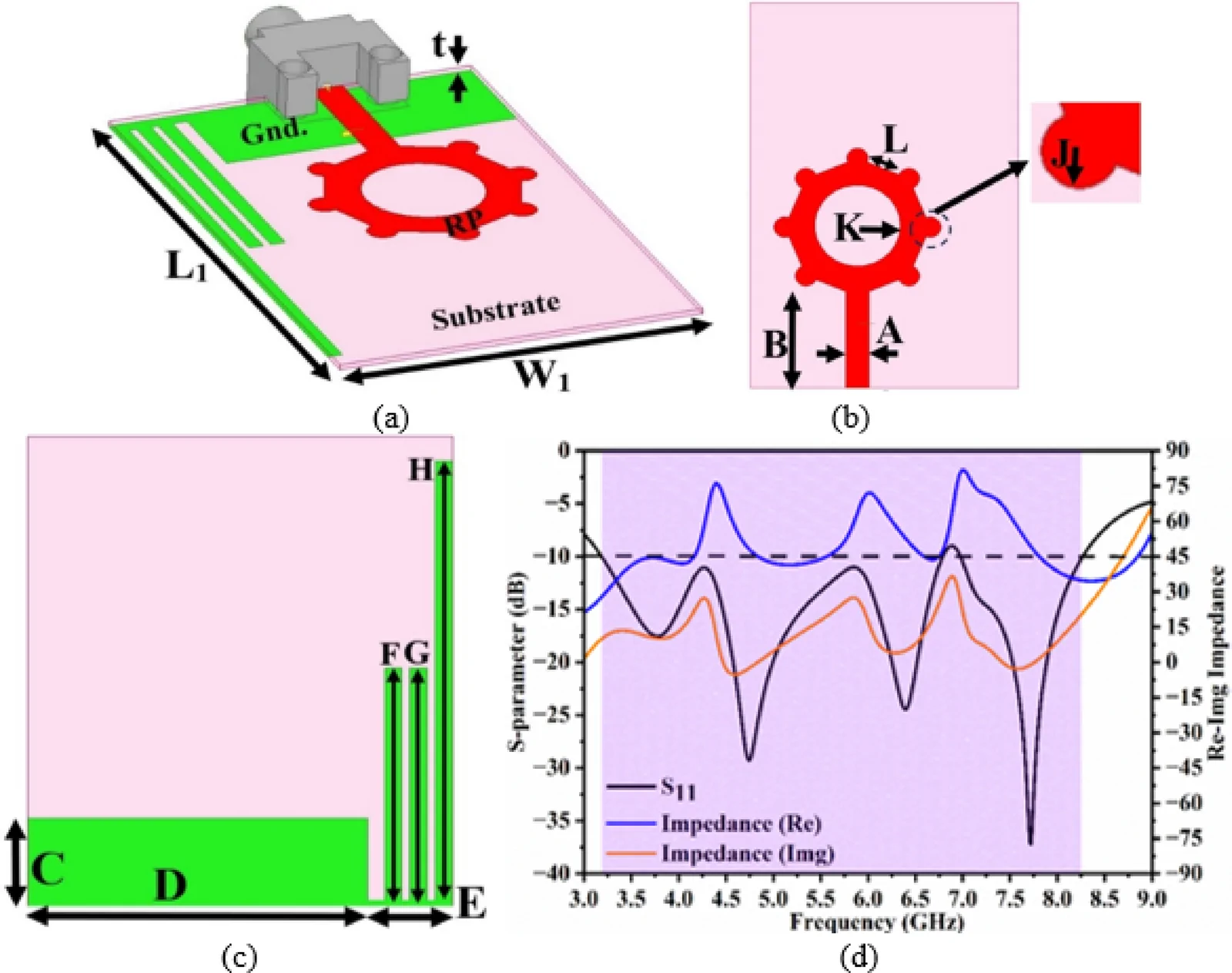
Proposed multiband antenna: (**a**) isometric view with dimensional parameters, (**b**) top layer, (**c**) bottom layer, and (**d**) S-parameter and impedance plots.

The multiband antenna plays a vital role in replacing several single-band antennas printed on the motherboard. This optimizes space and enables the integration of more complex circuits for different purposes. Patch antennas are widely used for this purpose because they can be easily printed and integrated with microwave integrated circuits (MICs). Figure 1 provides insight into the proposed multiband antenna, which generates a wide impedance bandwidth covering multiple applications.

Figure 1(a) shows the isometric view of the proposed single-port antenna, where an FR4 substrate is used, with the radiating patch (RP) and ground plane (Gnd) occupying two opposite surfaces. The overall dimensions of the antenna with the simulation-modeled SMA connector are L_1_ × W_1_ = 25 × 38 mm², with a substrate thickness of t = 1.60 mm. The front view of the radiating patch is shown in Fig. 1(b), with a circular ring patch embedded with seven circular cuts of radius J placed on the circumference. The octagonal patch, with each side of L mm, is connected to a 50 Ω microstrip feed line of dimensions A × B mm². A novel ground is printed on the opposite surface of the patch, with a rectangular ground area of C × D mm² attached to three rectangular strips, which improves impedance matching. Figure 1(d) shows the S-parameter and impedance plots. The impedance bandwidth generated by the antenna records a lower cutoff frequency of 3.20 GHz and an upper cutoff frequency of 8.27 GHz. Figure 1(d) also records the impedance graph, with the real and imaginary parts plotted with respect to frequency. The four resonances at 3.78 GHz, 4.74 GHz, 6.39 GHz, and 7.72 GHz record respective input impedances of (44.32 + j11.26) Ω, (48.31 − j2.92) Ω, (53.0 + j5.40) Ω, and (49.19 − j1.10) Ω. The proposed antenna is well suited for 5G, Wi-Fi 5, and Wi-Fi 6E applications, with the optimized dimensions recorded in Table 1.

**Table 1:** Optimized design parameters of the proposed antenna.

Parameter	L_s_	W_s_	A	B	C	D	E
Value (mm)	60	60	2.2	9.5	7.5	20	7.0
Parameter	F	G	H	I	J	K	L
Value (mm)	18.5	18.5	36	1.5	1	4	5.2

**Fig. 2:**
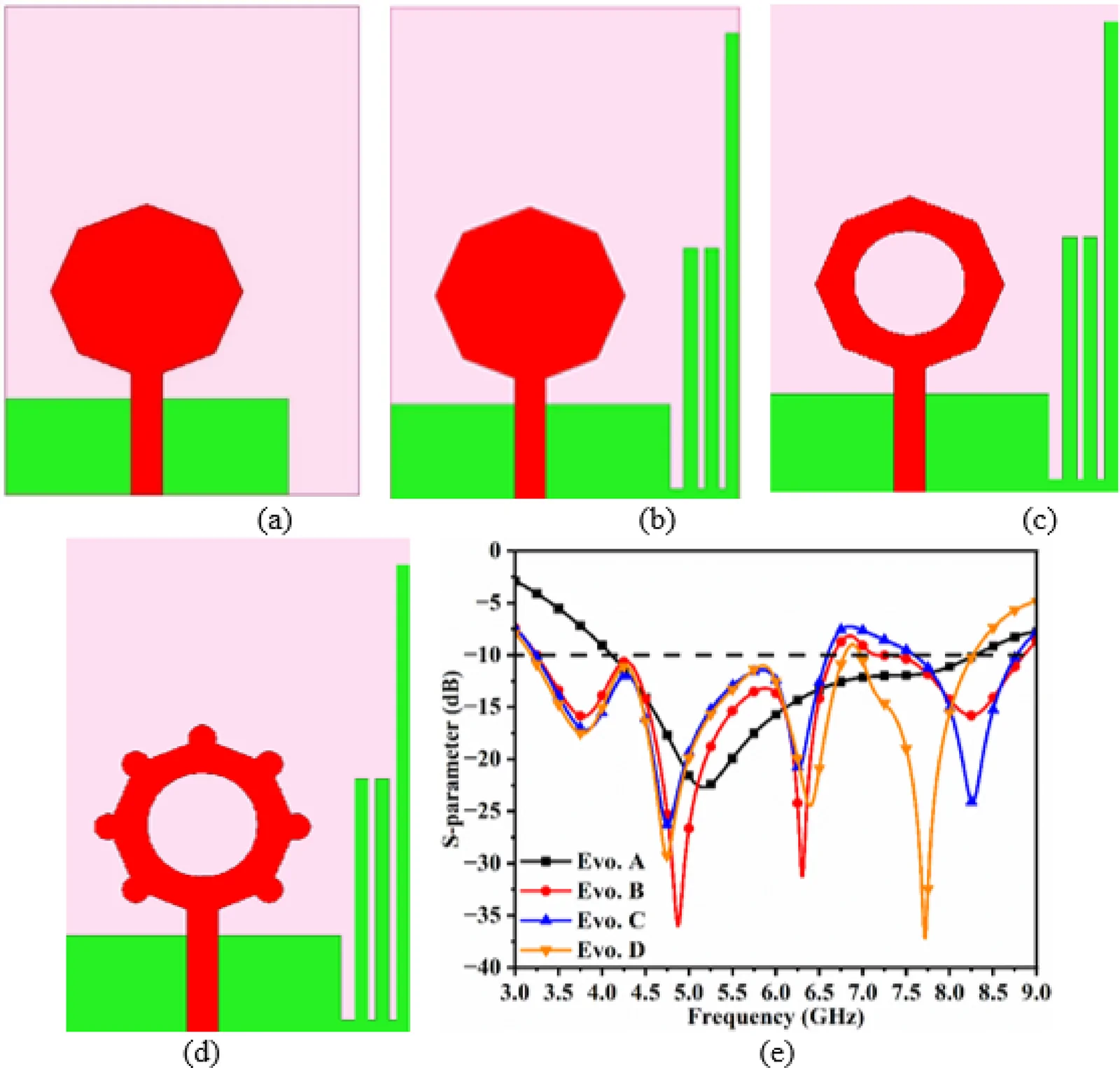
Evolution of the multiband antenna: (**a**) Evolution A (Evo. A), (**b**) Evolution B (Evo. B), (**c**) Evolution C (Evo. C), (**d**) Evolution D (Evo. D), and (**e**) S-parameter comparison of the evolutions.

The antenna shown in Fig. 1, generating a wider impedance bandwidth in its final version, is obtained by iterating the design through the four evolutions shown in Fig. 2. Evolution A comprises an octagonal-shaped patch geometry supported by a rectangular ground, as shown in Fig. 2(a). It records a bandwidth of 4.14–8.30 GHz with one resonance centered at 5.16 GHz. Evolution A consists of a basic octagonal radiating patch with a rectangular partial ground plane. The octagonal geometry provides a larger current path compared with a simple rectangular monopole, and its multiple edges help excite a fundamental resonance. However, the current path is still relatively simple, so the antenna mainly produces a limited resonant response. The partial ground plane introduces fringing fields between the feed line, patch, and ground edge, but the impedance matching is not sufficient over the complete desired range. Therefore, this stage supports only a restricted operating bandwidth with a dominant resonance around the mid-band. The next iteration is Evolution B, in which three slits are added to the ground. Two additional impedance-matched resonances are achieved, centered at 4.86 GHz and 6.29 GHz, respectively, but the matching deteriorates above 7.0 GHz. These slits disturb the ground current distribution and create additional current paths. Electromagnetically, the slits behave as inductive-capacitive discontinuities. The length of the slit increases the effective current path, while the narrow gap between the slit edges introduces capacitance. This combined LC effect produces additional resonant modes and improves impedance matching near the lower and middle frequency regions. As a result, extra resonances appear around the 5G/WLAN region. However, because the ground current becomes strongly perturbed, matching at the higher-frequency region may deteriorate. Thus, the ground slits improve multiband behavior but alone do not provide uniform wideband performance. Evolution C is achieved by etching a circular slot at the center of the octagonal patch, which improves impedance matching between 7.60 GHz and 8.76 GHz. This evolution changes the surface-current distribution on the radiator itself. The central slot forces the current to flow around the slot rather than directly across the patch, thereby increasing the effective electrical length of the radiating path. At the same time, the circular slot introduces capacitive loading between the inner and outer edges of the patch. This improves impedance matching at higher frequencies and helps excite another resonant mode. The ring-like current path formed around the circular slot is responsible for bandwidth enhancement in the upper part of the operating band. Therefore, Evolution C improves the high-frequency response compared with the previous stage. The final version of the antenna, Evolution D, is formed by adding seven smaller circles at the corners of the octagonal radiating patch, which provides good impedance matching, as shown in Fig. 2(e). These small circular perturbations further modify the edge current distribution and increase the perimeter of the radiating structure. Since high-frequency currents are mainly concentrated near the patch edges, these circular cuts strongly influence the higher-order resonant modes. The small circular cuts act as distributed capacitive loading points and create multiple localized current paths. This improves impedance matching across the desired frequency range and helps merge the nearby resonances into a wider operating band. Therefore, the final geometry provides a better combination of lower-band, middle-band, and upper-band resonances.

Physically, the final multiband behavior is obtained because the antenna contains several resonating sections: the octagonal outer boundary, the circular ring slot, the peripheral circular cuts, the microstrip feed line, and the modified partial ground plane with slits. The octagonal patch mainly supports the fundamental resonance, the ground slits improve the lower- and middle-band matching, the central circular slot enhances the upper-frequency resonance, and the small circular cuts improve the edge-current tuning and broadband impedance matching. The interaction of these multiple current paths produces the final wide impedance bandwidth of approximately 3.20–8.27 GHz for the single-port antenna, making it suitable for 5G sub-6 GHz, Wi-Fi 5, and Wi-Fi 6E applications.

## Four-port MIMO antenna design and isolation mechanism

**Fig. 3:**
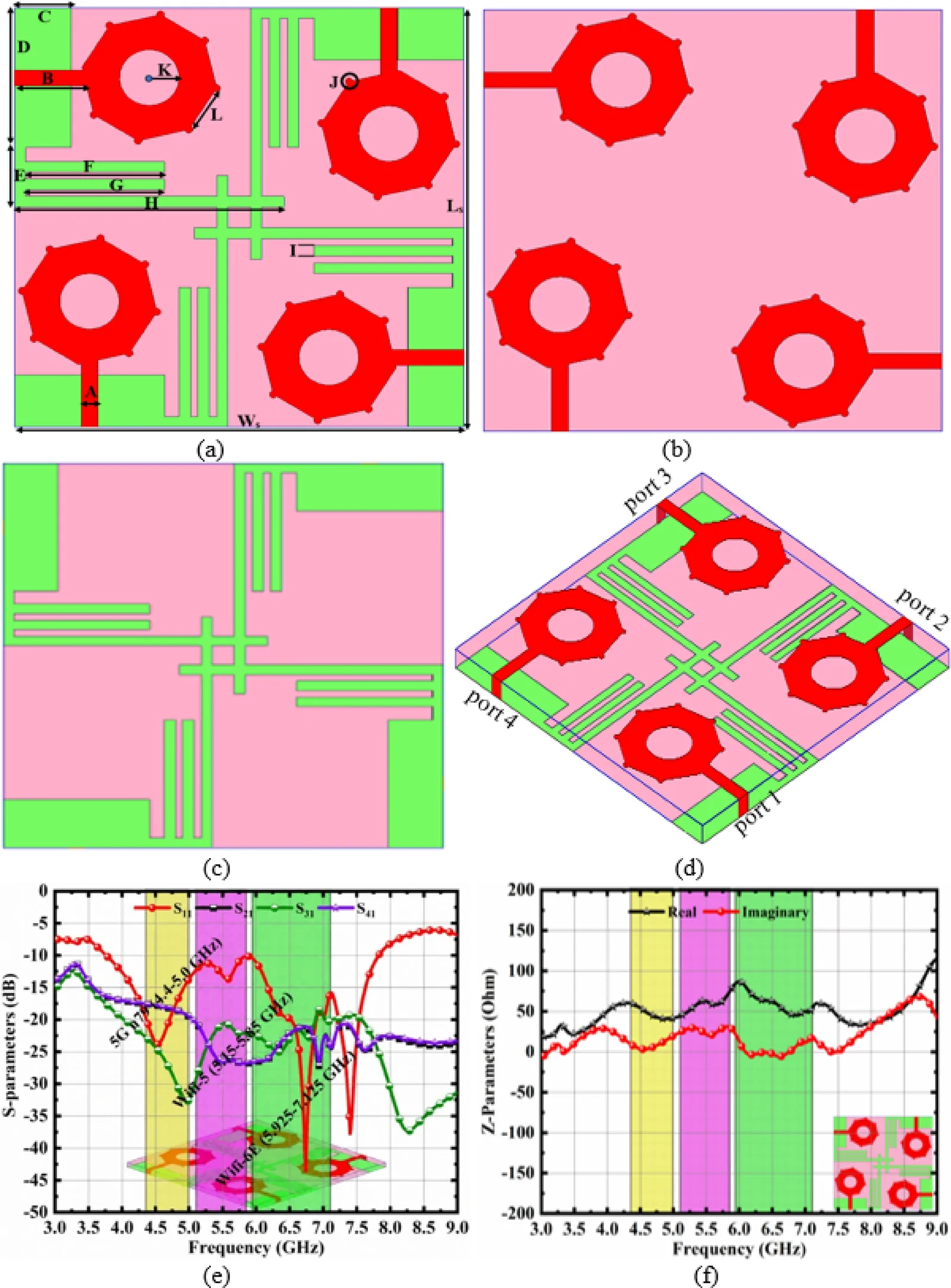
Proposed MIMO antenna: (**a**) dimensional parameters, (**b**) top layer, (**c**) bottom layer, (**d**) 3D isometric view, (**e**) four-port MIMO antenna S-parameter plot, and (**f**) input-impedance plot.

Figure 3 presents the structural configuration, layer-wise layout, 3D view, S-parameter response, and input-impedance characteristics of the proposed four-port MIMO antenna. Figure 3(a) shows the proposed design structure with its isometric view, top layer, and ground-layer details. A low-cost FR4 substrate has been used with dielectric constant (εr = 4.4), thickness h = 1.6 mm, and loss tangent tan δ = 0.02, with overall dimensions of 60 × 60 mm². The top layer of the design is formed as an octagonal ring-type structure with a partial ground plane and conventional microstrip feeding. The dimensional parameters of the design are tabulated in Table 1. Figure 3(b) shows the radiating patches, which are arranged orthogonally to achieve spatial diversity. This arrangement not only preserves the impedance bandwidth generated by each radiating patch but also maintains isolation between them. Figure 3(c) shows the commonly connected ground. It addresses the issue of disconnected ground, where a direct split in the ground can artificially enhance port isolation because there is no direct current coupling. However, in the proposed design, each ground section is interconnected and provides a common-reference ground^38^.

In the proposed MIMO antenna, the connected ground provides a practical common-reference ground, which is more suitable for real wireless devices compared with completely separated ground planes. The narrow connecting strips and optimized slits control the return-current path and suppress unwanted surface current propagation between adjacent radiating elements. This helps in improving impedance matching over the wide operating band while keeping mutual coupling low.

Unlike conventional DGS-based designs, where defects are etched in the ground plane mainly to disturb current flow, the proposed connected ground preserves electrical continuity while using strips and slits to guide and restrict the current distribution. Unlike neutralization-line techniques, no additional coupling line is introduced between antenna elements. Similarly, unlike parasitic-element-based decoupling, the proposed design does not require extra resonant elements between the ports. Therefore, the connected ground structure offers a simpler, compact, and practically integrable solution for achieving wideband impedance matching and acceptable isolation in a four-port MIMO antenna.

The 3D structure is shown in Fig. 3(d), with each antenna element excited by a 50 Ω lumped port, generating the S-parameters plotted in Fig. 3(e). The S-parameters of the proposed design are depicted in Fig. 3(e), which shows the reflection and transmission coefficients. The reflection coefficient shows multiple resonance dips in the frequency band of 3.3–7.7 GHz. The design covers the − 10 dB impedance bandwidth for all targeted specifications. For instance, the 5G n79 band (4.4–5.0 GHz), Wi-Fi 5 (5.15–5.85 GHz), and Wi-Fi 6E (5.925–7.125 GHz) are covered with excellent impedance matching, as shown by the red curve. Moreover, transmission coefficients are plotted in terms of S21, S31, and S41. It can be observed that all values are below − 20 dB. S21 and S41 follow almost the same pattern because the port-2 and port-4 elements are adjacent to port 1. Due to the placement of the port-3 element, S31 shows improved isolation in the 5G n79 band and only slight deterioration, within the specified limits, in the other two bands. Further, Fig. 3(f) shows the variation of the real and imaginary parts of the input impedance of the proposed antenna versus frequency, ranging from 3 GHz to 9 GHz. For a well-matched antenna, the real part should be close to 50 Ω, and the imaginary part should be near 0 Ω within the operating frequency bands.

**Fig. 4:**
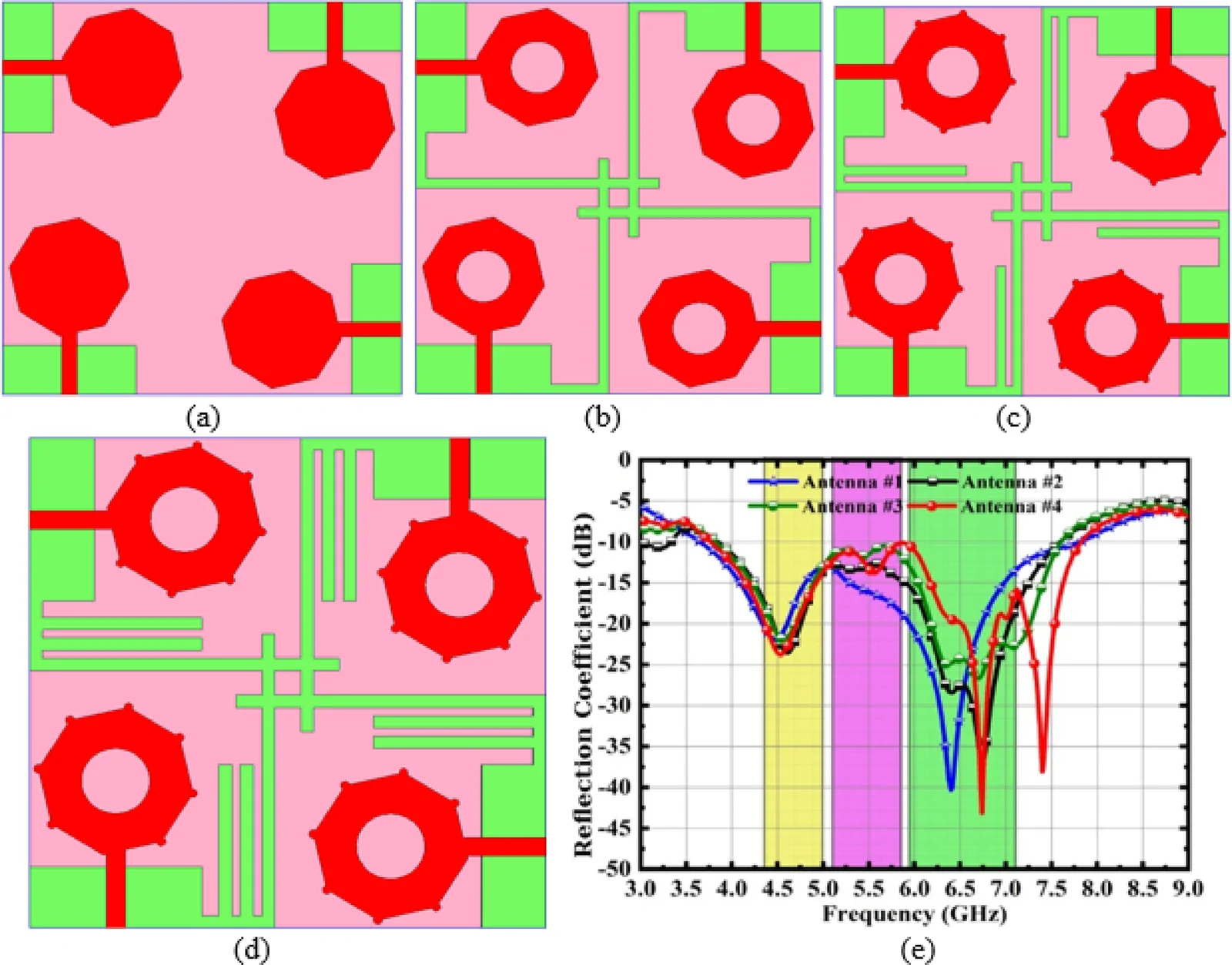
Proposed MIMO antenna evolution: (**a**) Antenna #1, (**b**) Antenna #2, (**c**) Antenna #3, (**d**) Antenna #4, and (**e**) S11 plot.

The shaded regions indicate the resonant bands where the antenna exhibits good impedance matching and operates efficiently. In these bands, the real impedance approaches 50 Ω, and the imaginary component crosses or stays near zero, confirming proper resonance conditions. Thus, this plot confirms that the antenna achieves good impedance matching at multiple frequency bands, highlighting its multiband operation and efficient energy transfer between the antenna and the feed line.

Further, the evolution of the proposed design can be visualized in Fig. 4. Figure 4(a), marked as Antenna #1, depicts four octagonal patches with individual partial ground planes arranged in orthogonal orientations. This arrangement exhibits two resonances in the 5G n79 and Wi-Fi 6E frequency bands while maintaining the − 10 dB impedance bandwidth from 3.3 to 7.7 GHz, as shown by the blue curve. It is also worth noting that each ground is self-isolated from the others and achieves high isolation; however, common-ground issues remain when the antenna is integrated with other devices on the same motherboard. In the next iteration, shown in Fig. 4(b) as Antenna #2, the partial grounds are connected with 1.5 mm wide transmission lines and show almost similar resonance variations in the 5G n79 frequency band but improved bandwidth in the Wi-Fi 6E frequency band, as shown by the black curve. In the next iteration, represented by Fig. 4(c), two additional transmission lines are attached to all the ground planes to improve the resonance characteristics of the proposed antenna design.

The isolation mechanism in the final MIMO antenna can be summarized as follows:

Step 1: Orthogonal placement of radiating elements.

The four octagonal ring-shaped elements are placed in orthogonal directions. This reduces field overlap and provides polarization diversity between adjacent ports.

Step 2: Spatial separation between ports.

The four elements are distributed around the 60 × 60 mm² substrate, which increases the physical separation between radiators and reduces direct near-field coupling.

Step 3: Controlled common ground connection.

The separate ground sections are connected using narrow strips. These strips provide a common reference ground but restrict uncontrolled current flow between ports.

Step 4: Ground-current path modification.

The connected ground modifies the return-current distribution and helps maintain impedance matching over the desired 3.3–7.7 GHz operating band.

Step 5: Introduction of open-ended ground slits.

Additional slits are inserted in the connected ground region. These slits act as current blockers and increase the electrical path length for coupled surface currents.

Step 6: Suppression of surface current coupling.

When one port is excited, and the other ports are terminated with 50 Ω loads, the slits confine most of the current near the excited element. Very weak current reaches the adjacent and opposite elements, resulting in reduced mutual coupling.

Step 7: Final high-isolation performance.

Due to the combined effect of orthogonal arrangement, controlled connected ground, and slit-based current suppression, the final Antenna #4 achieves isolation better than − 17.5 dB across the operating band while maintaining a wide impedance bandwidth.

Thus, the physics behind the MIMO evolution in Fig. 4 is based on controlling the surface-current distribution. The initial orthogonal placement reduces polarization coupling, the connected ground provides practical integration, and the optimized slotted ground suppresses unwanted current propagation between ports. As a result, the final MIMO antenna achieves wideband operation, improved impedance matching, and high inter-port isolation suitable for 5G n79, Wi-Fi 5, and Wi-Fi 6E applications.

**Fig. 5:**
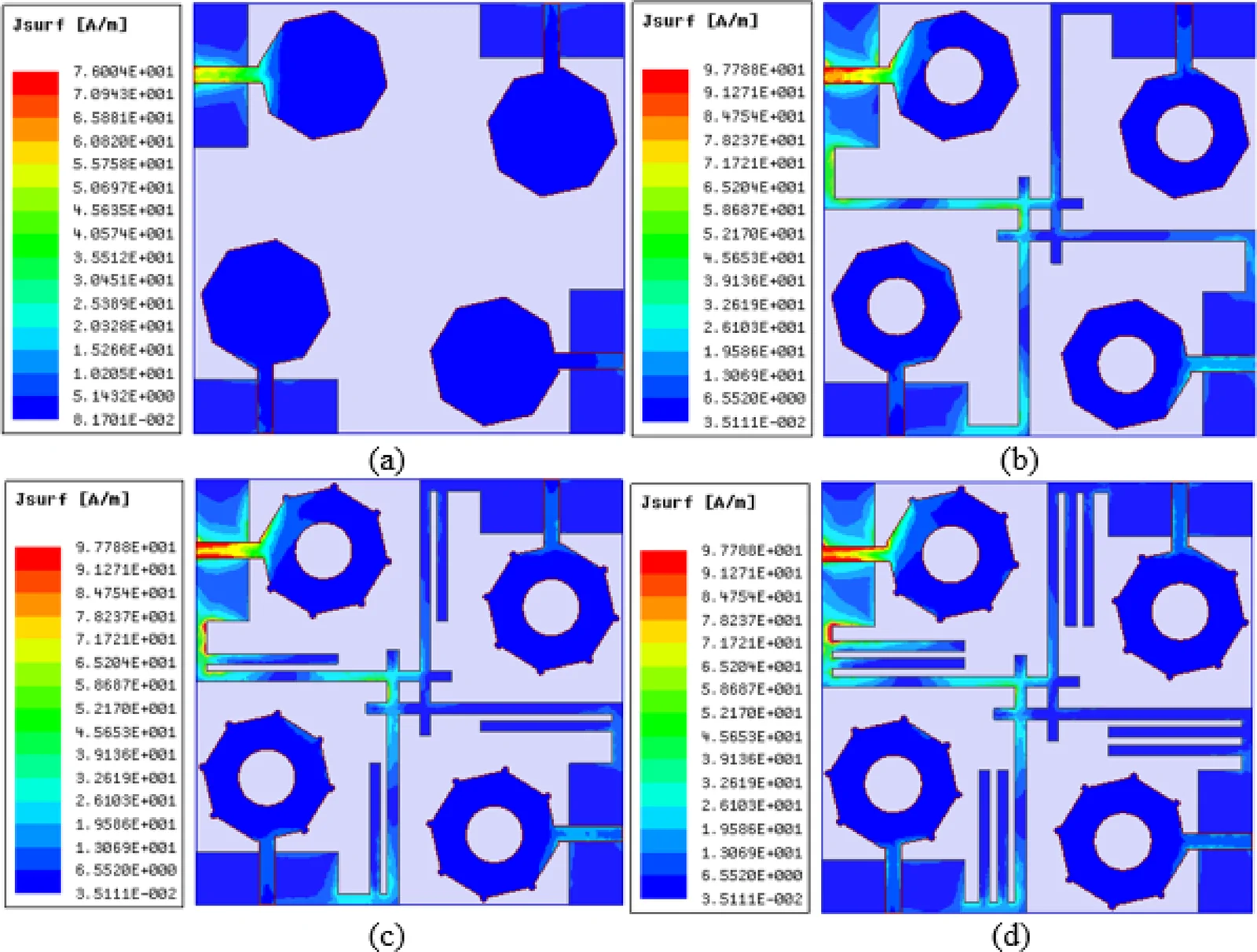
Proposed MIMO antenna evolution surface-current-density (SCD) response: (**a**) Antenna #1, (**b**) Antenna #2, (**c**) Antenna #3, and (**d**) Antenna #4.

From Fig. 4, it is observed that Antenna #3 shows similar behavior but at the cost of lower impedance matching, as shown by the green curve. However, Antenna #4, Fig. 4(d), with the additional transmission-line and ground-slit arrangement, outperforms all previous iterations in terms of multiple resonance frequencies, with a maximum bandwidth of approximately 4 GHz and excellent impedance matching of − 43 dB in the Wi-Fi 6E frequency band, as shown by the red curve. The above-mentioned resonance behavior of the proposed Antenna #4 can be justified by the surface-current distribution depicted in Fig. 5. Figure 5(a) shows almost no coupled current on the other radiating elements when port 4 is excited. The direct split in the ground achieves direct isolation enhancement because there is no current coupling. However, this issue has been discussed above, and the commonly connected ground with four slits, shown in Fig. 5(b), provides additional current paths but does not sufficiently reduce coupling. In addition, four more open-ended slits are placed adjacent to the commonly connected slits, serving as additional current paths that enhance isolation.

**Fig. 6:**
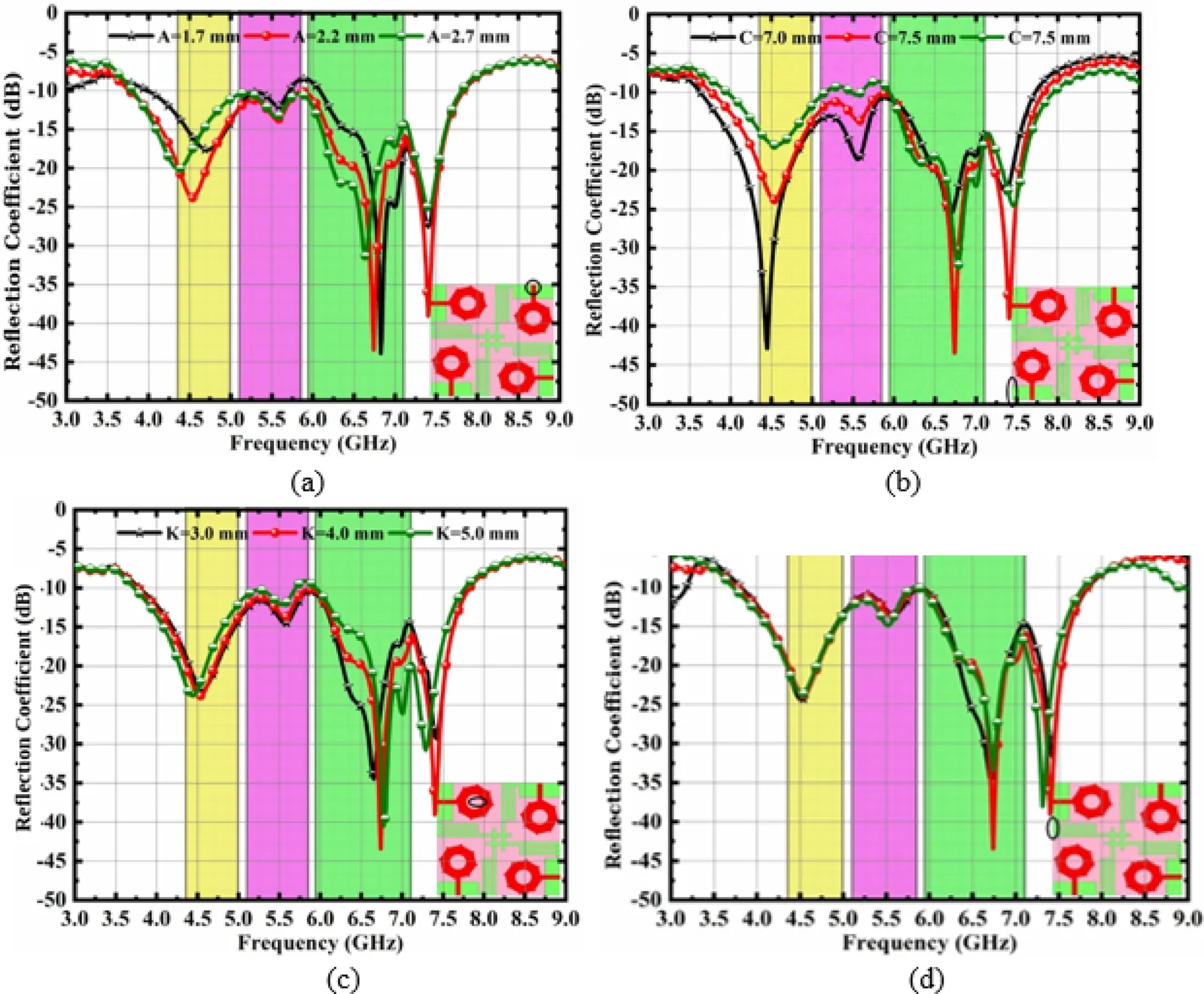
Proposed MIMO antenna parametric analysis: (**a**) feed line width (A), (**b**) ground-plane length (C), (**c**) radius of the circular cut (K), and (**d**) extension of ground-plane length (E).

In the final version, Antenna #4, Fig. 5(d), four additional open-ended slits are added in the ground to reduce inter-element interference and increase isolation. The SCD analysis is carried out by exciting port 4, while the remaining ports are terminated with a matched impedance of 50 Ω. It is observed that the proposed antenna employs multiple resonating elements, including a patch, a partial ground plane, and microstrip lines. High surface current is observed at the feed location and near the patch edges, while the current density decreases in farther regions due to power division or mutual coupling. This current-distribution behavior suggests that the antenna structure exhibits wideband operation.

Next, the impact of various dimensional parameters on the resonance characteristics is studied using parametric optimization, as shown in Fig. 6. First, impedance matching is strongly affected by the width of the microstrip feed line, as depicted in Fig. 6(a). The feed line width A is varied by ± 0.5 mm, and A = 2.2 mm produces the maximum number of resonating peaks with excellent − 10 dB impedance matching in the required frequency band of operation compared with the other two feed line widths, 1.7 mm and 2.7 mm. Next, the length of the ground plane C is optimized to achieve the required matching in the targeted applications. Again, a ± 0.5 mm variation is applied to the parameter, and C = 7.5 mm is found to be the optimized value for the best impedance matching in the specified frequency bands, as depicted in Fig. 6(b). In the next parameter, the radius of the circular cut K is optimized with a variation of ± 1 mm, which strongly affects the impedance matching and bandwidth in the Wi-Fi 6E frequency band, as shown in Fig. 6(c). It can be observed that for all values of K, the resonance curves follow almost the same pattern in the 5G n79 band, and the maximum bandwidth with excellent impedance matching is achieved in the Wi-Fi 6E frequency band at the optimized value of K = 4 mm. Finally, the extended length of the ground plane E is optimized with a variation of ± 1 mm, and the design exhibits almost the same resonance characteristics across the entire band, except for excellent matching at E = 7.0 mm, as shown in Fig. 6(d).

## Results and discussion

This section presents the simulated and measured validation of the proposed four-port MIMO antenna. The discussion first examines the fabricated prototype and S-parameter performance, followed by the evaluation of MIMO diversity metrics, gain, radiation efficiency, radiation patterns, and SAR compliance. These analyses collectively verify the antenna’s bandwidth, isolation, diversity behavior, radiation stability, and safety for practical 5G NR n79, Wi-Fi 5, and Wi-Fi 6E applications.

### Fabrication and S-parameter measurement

**Fig. 7:**
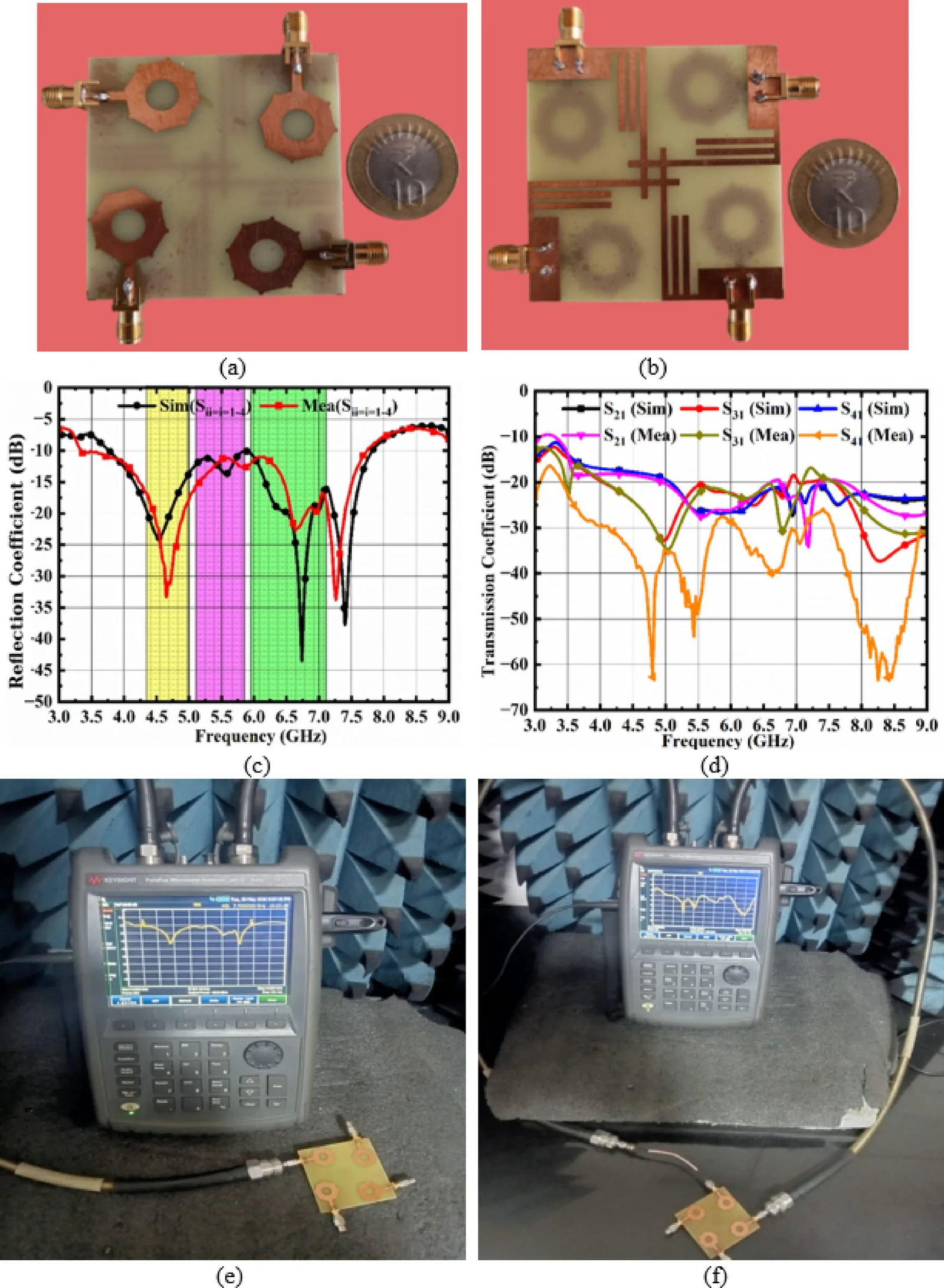
Proposed MIMO antenna: (**a**) prototype top view, (**b**) prototype bottom view, (**c**) reflection coefficient results, (**d**) transmission coefficient results, (**e**) VNA screenshot for measured Sii (i = 1–4), and (**f**) measured S41.

Figure 7 presents the fabricated prototype, simulated and measured reflection/transmission coefficients, and VNA measurement screenshots of the proposed MIMO antenna. The top and bottom layers of the fabricated prototype of the proposed design are shown in Fig. 7(a, b). The corresponding comparison of the simulated and measured resonance characteristics is illustrated in Fig. 7(c, d). The simulated and measured reflection coefficients show good agreement, except for some deterioration in the measured impedance matching. Figures 7(e, f) show screenshots of the prototype connected to the VNA for reflection-coefficient and transmission-coefficient measurements. The Keysight FieldFox Microwave Analyzer model N9935C, with a maximum measurement capability of 10 GHz, is used in this work.

The minor differences between simulation and measurement are due to SMA connectors and solder joints adding parasitic capacitance and inductance, which are not modeled in simulations. Imperfect soldering or connector misalignment can also change the input impedance and shift the reflection coefficient. Similarly, the simulated and measured transmission coefficients, S21, S31, and S41, show very good agreement.

### MIMO diversity performance

**Fig. 8:**
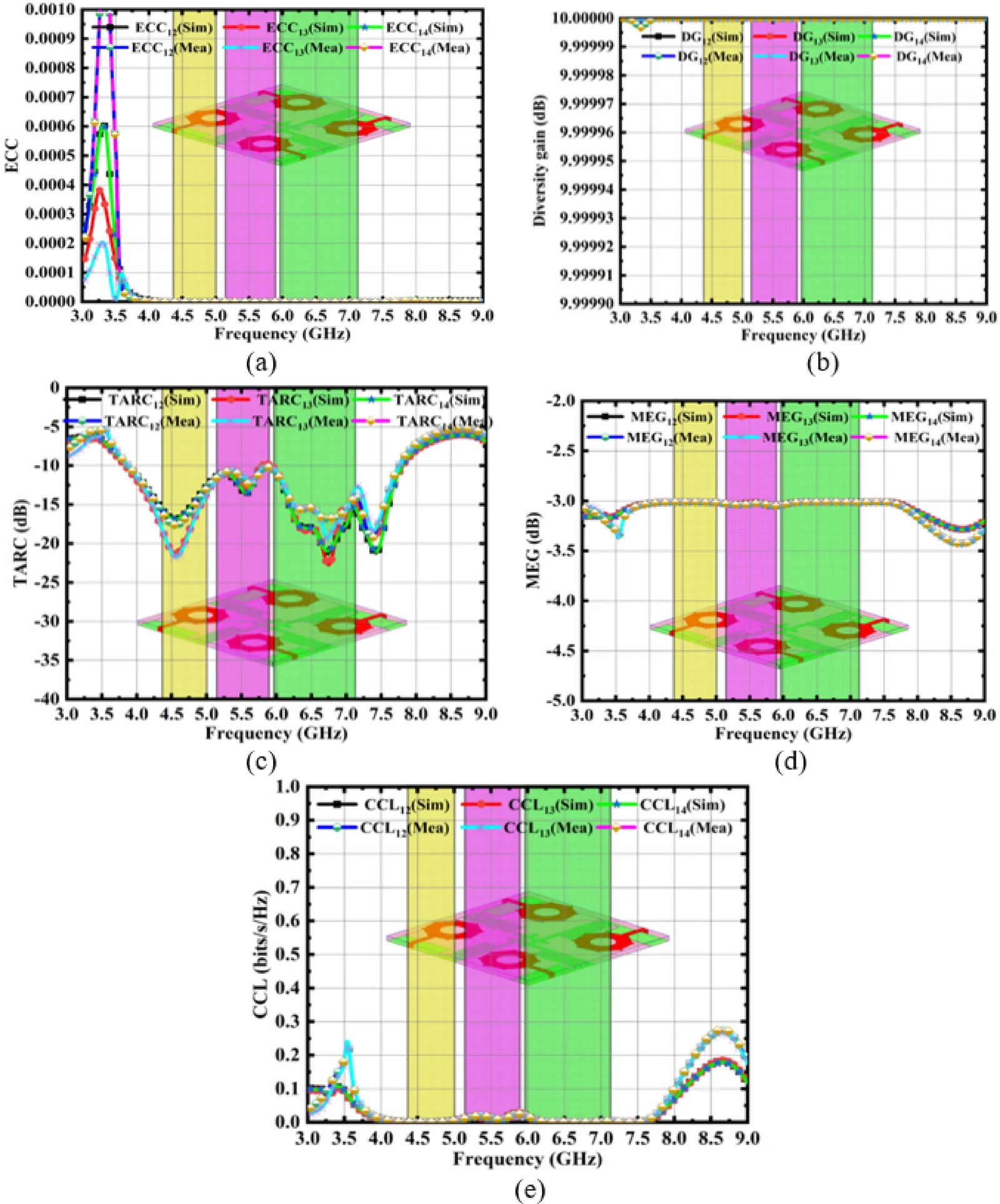
Proposed MIMO antenna diversity metrics: (**a**) ECC, (**b**) DG, (**c**) TARC, (**d**) MEG, and (**e**) CCL.

The MIMO behavior of the proposed antenna is verified by analyzing the diversity metrics, including envelope correlation coefficient (ECC), diversity gain (DG), total active reflection coefficient (TARC), mean effective gain (MEG), and channel capacity loss (CCL). Figure 8 presents the corresponding simulated and measured results for these metrics, confirming the diversity performance of the proposed MIMO antenna.

#### ECC

The ECC establishes the correlation between the radiating elements in the MIMO configuration and can be expressed using Eq. (1) for two ports. Similar equations can be formulated for a greater number of elements. It is observed that the simulated and measured values are in good agreement, and all values are well below 0.0001 in the targeted frequency bands, as depicted in Fig. 8(a).

1 $$\:{\rho\:}_{s}=\frac{{\left|{S}_{11}^{*}{S}_{12}+{S}_{21}^{*}{S}_{22}\right|}^{2}}{\left(1-{\left|{S}_{11}\right|}^{2}-{\left|{S}_{21}\right|}^{2}\right)\left(1-{\left|{S}_{22}\right|}^{2}-{\left|{S}_{21}\right|}^{2}\right)}$$

where S11 and S22 are the input reflection coefficients of the two ports, and S12 is the coupling between the ports. The value of ECC should be as low as possible to ensure low correlation and low mutual coupling between the radiating elements.

#### DG

Diversity gain quantifies the improvement obtained from diversity reception, as expressed in Eq. (2). Figure 8(b) shows that the DG is nearly 10 dB in the desired frequency range.

2 $$\:DG=10\sqrt{1-{\left|{\rho\:}_{s}\right|}^{2}}$$

#### TARC

TARC represents the ratio of total reflected power to total incident power when the ports are excited simultaneously, and it can be expressed using Eq. (3). The variations of TARC with frequency are shown in Fig. 8(c). The simulated and measured values are very close, and the values lie below − 10 dB, indicating that most of the incident power is accepted by the antenna system. Consequently, this further confirms that the proposed four-port MIMO antenna is suitable for MIMO operation.

3 $$TARC = \sqrt {\frac{{\left( {S_{{11}} + S_{{12}} } \right)^{2} + \left( {S_{{21}} + S_{{22}} } \right)^{2} }}{2}}$$

#### MEG

Mean effective gain is another crucial diversity-performance metric for describing MIMO antennas in wireless channels. In a multipath fading environment, MEG evaluates the ability of each antenna element to receive electromagnetic signals. The variations of the simulated and measured values are depicted in Fig. 8(d) and expressed in Eq. (4).

4 $$\begin{gathered} MEG_{i} = 0.5\left[ {1 - \left| {S_{{ii}} } \right|^{2} - \left| {S_{{ij}} } \right|^{2} } \right] \hfill \\ MEG_{j} = 0.5\left[ {1 - \left| {S_{{jj}} } \right|^{2} - \left| {S_{{ij}} } \right|^{2} } \right] \hfill \\ MEG = \frac{{MEG_{i} }}{{MEG_{j} }} \hfill \\ \end{gathered}$$

#### CCL

The last parameter discussed is CCL. A CCL below 0.1 bits/s/Hz indicates acceptable channel-capacity performance, as shown in Fig. 8(e).

### Gain, efficiency, and radiation characteristics

**Fig. 9:**
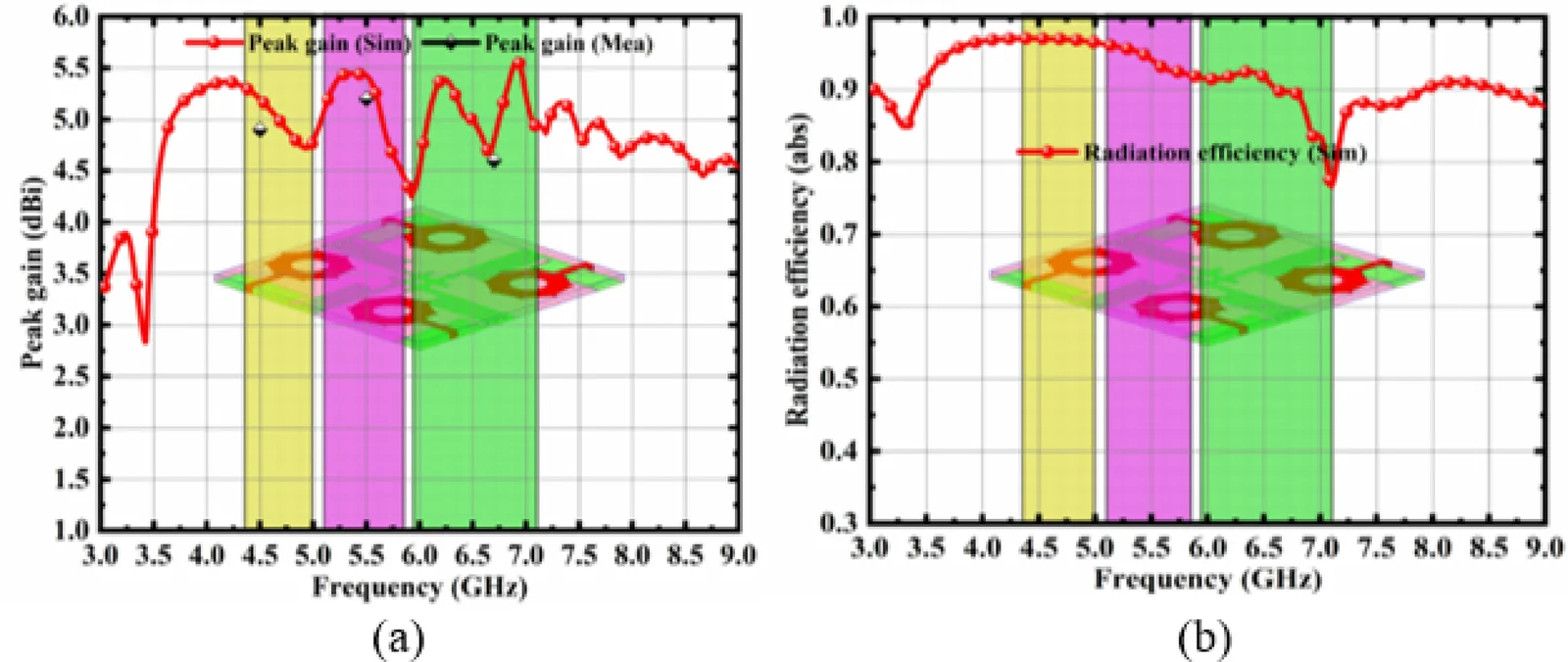
Far-field analysis: (**a**) simulated and measured peak realized gain (dBi) and (**b**) radiation efficiency.

Figure 9 presents the far-field gain and radiation-efficiency performance of the proposed MIMO antenna over the investigated frequency range. The simulated and measured peak realized gain as a function of frequency, ranging from 3 GHz to 9 GHz, is illustrated in Fig. 9(a). The red curve with circular markers represents the simulated peak gain, while the measured peak gain is represented by the corresponding measured curve in the figure. The antenna demonstrates a relatively stable peak gain of around 4.5–5.5 dBi across its targeted operating bands, with highlighted regions indicating the main operational frequency ranges: approximately 4.4–5.0 GHz, 5.1–5.9 GHz, and 6.1–7.1 GHz. The small differences between simulated and measured results are within acceptable limits, confirming good agreement and validating the antenna’s gain performance over multiple bands suitable for 5G NR n79, Wi-Fi 5, and Wi-Fi 6E wireless applications. Similarly, the simulated radiation efficiency is shown in Fig. 9(b). The analysis shows stable radiation efficiency of approximately 80%–96% in the specified frequency bands, with a minor dip at the edge of the Wi-Fi 6E frequency band due to factors such as mutual coupling between elements, surface-wave losses, and dielectric losses.

**Fig. 10:**
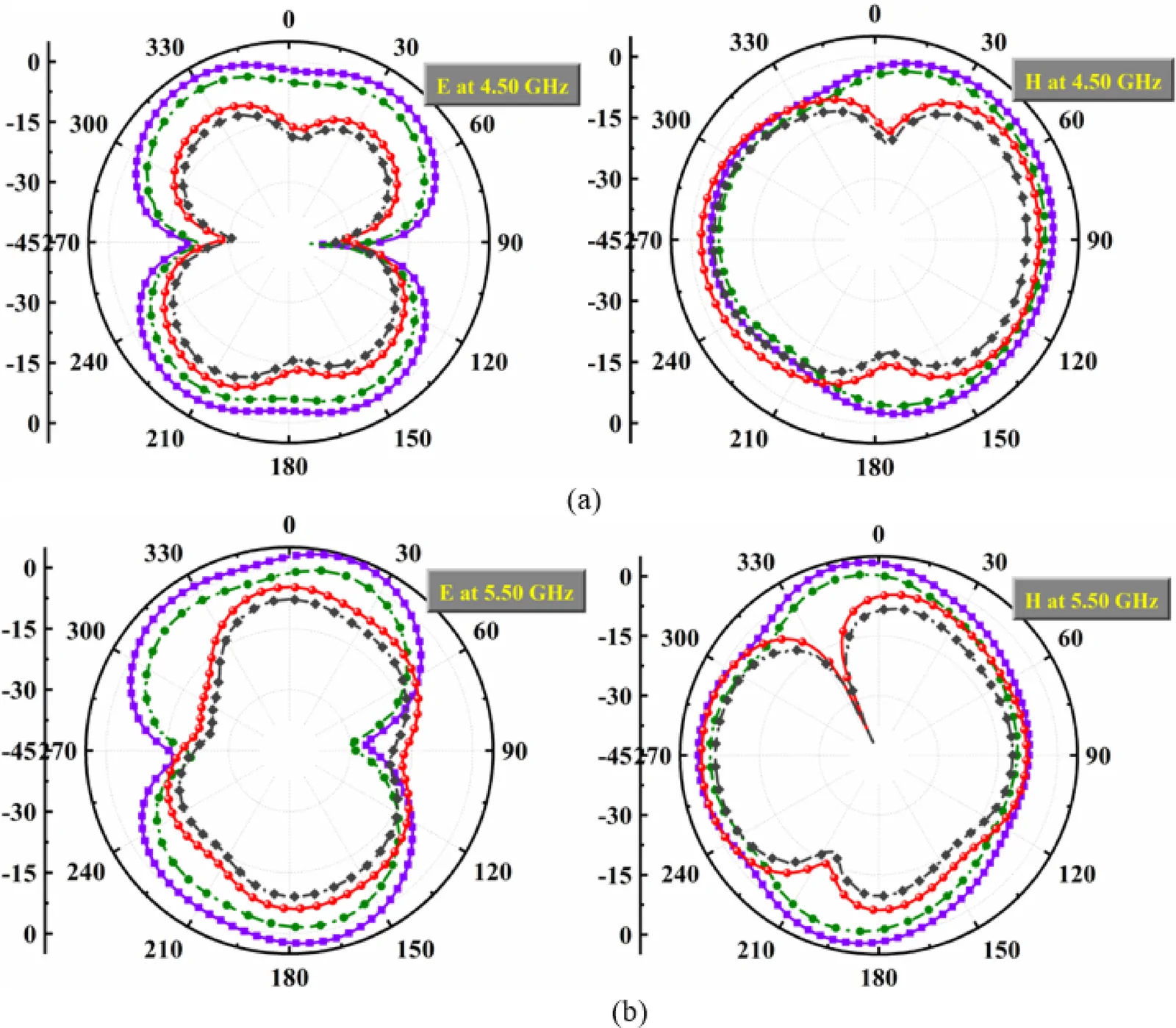

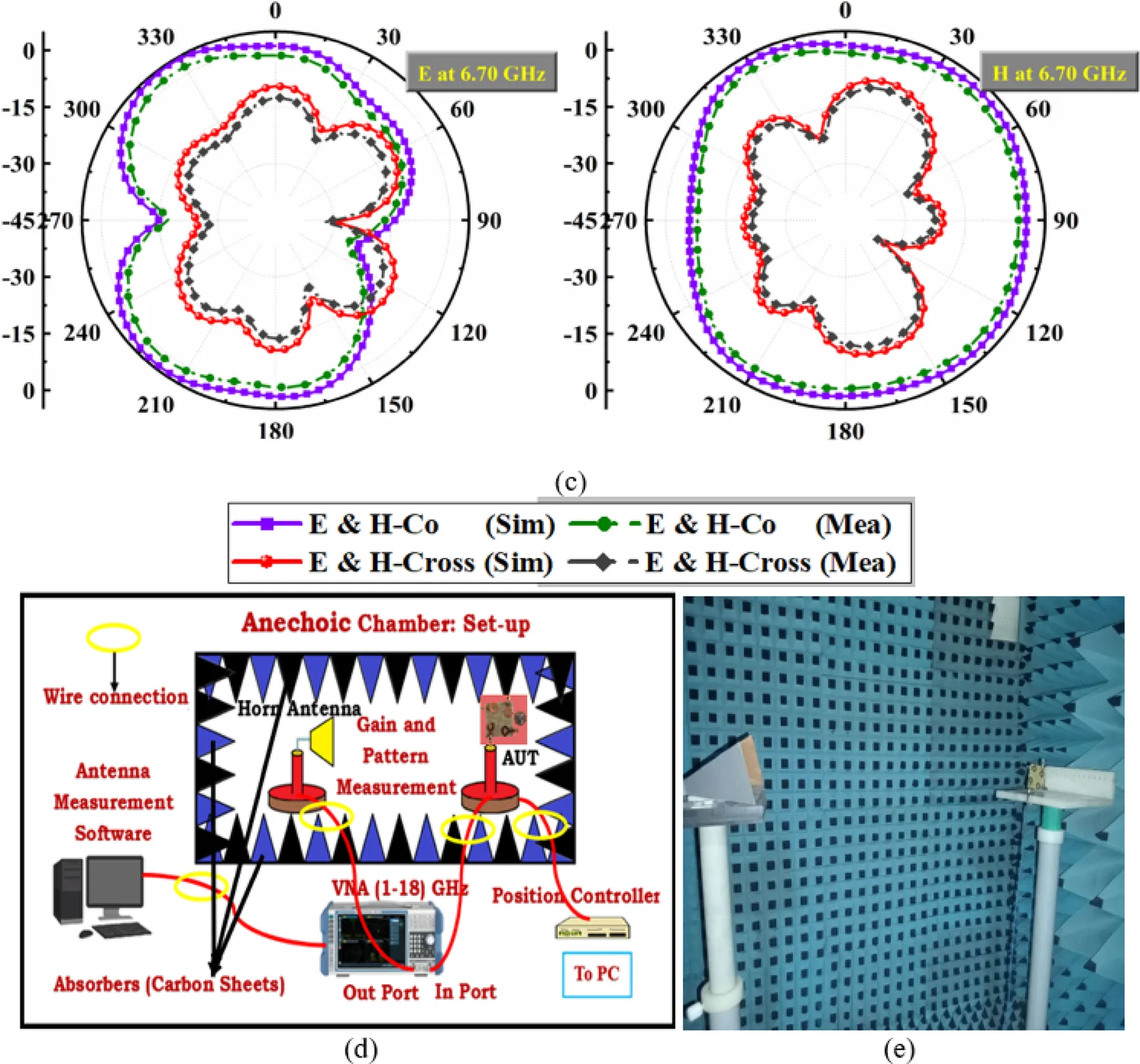
Proposed MIMO antenna: (**a**–**c**) 2D radiation responses, (**d**) chamber setup for the measurement process, and (**e**) prototype placed inside the anechoic chamber.

Moreover, the normalized 2D radiation patterns in terms of co-polarization and cross-polarization for both the E- and H-planes are shown in Fig. 10(a–c), and the measurement setup is illustrated in Fig. 10(d, e). The simulated and measured curves show very close agreement, with acceptable cross-polarization discrimination. The co-polarized pattern is nearly omnidirectional, providing uniform gain and stable radiation across the angular range.

The radiation patterns presented in Fig. 10 show that both the E-plane and H-plane patterns exhibit nearly similar behavior at the operating frequencies. The simulated and measured radiation patterns are also in close agreement, confirming the accuracy of the fabricated prototype and validating the antenna design. The minor variations observed between the measured and simulated results may be attributed to fabrication tolerances, connector losses, soldering effects, and measurement-environment imperfections. Overall, the consistency between the E- and H-plane patterns, along with the good agreement between simulated and measured data, demonstrates stable radiation performance and confirms the suitability of the proposed antenna for practical wireless communication applications. Figure 10d, e show the schematic and practical anechoic-chamber measurement setup, where a horn antenna is used to characterize the proposed four-port MIMO antenna.

**Fig. 11:**
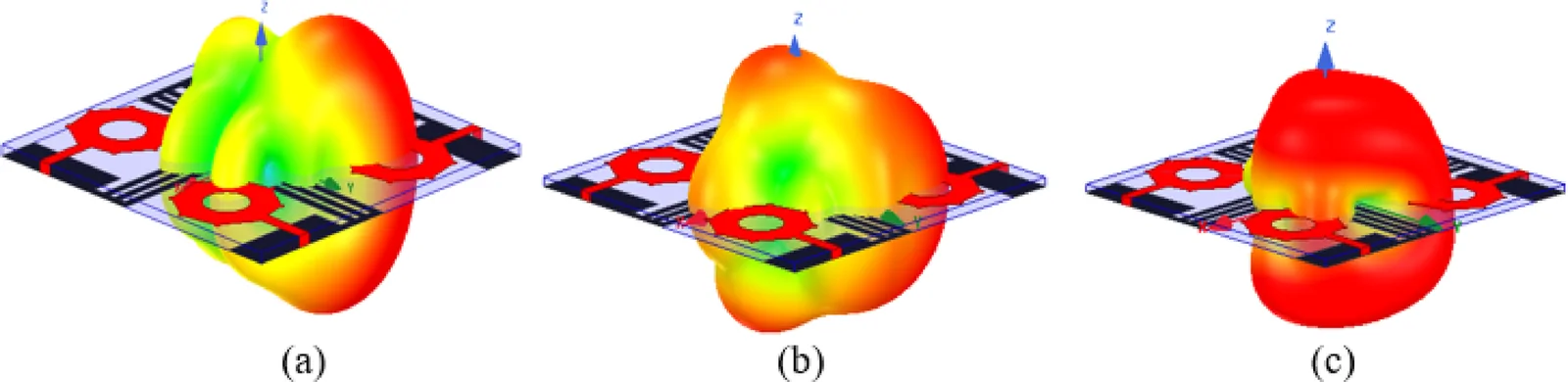
Proposed MIMO antenna 3D radiation at (**a**) 4.50 GHz, (**b**) 5.50 GHz, and (**c**) 6.70 GHz.

The 3D gain polar plots at the resonant frequencies are shown in Fig. 11. It can be observed that the gain is concentrated in the yz-plane at 4.50 GHz, as compared with the other directions; a similar pattern can be observed at 5.50 GHz. However, at 6.70 GHz, the radiation concentration is observed in multiple planes, showing nearly omnidirectional behavior. Figure 10 presents the 2D radiation patterns in the two principal planes, xy and yz, which are orthogonal and represent the E- and H-plane behavior of the antenna. Figure 11 provides further insight into the radiation patterns at the application resonant frequencies, with maximum peak gain observed around the upper and lower lobes.

### SAR analysis and human-head phantom validation

**Fig. 12:**
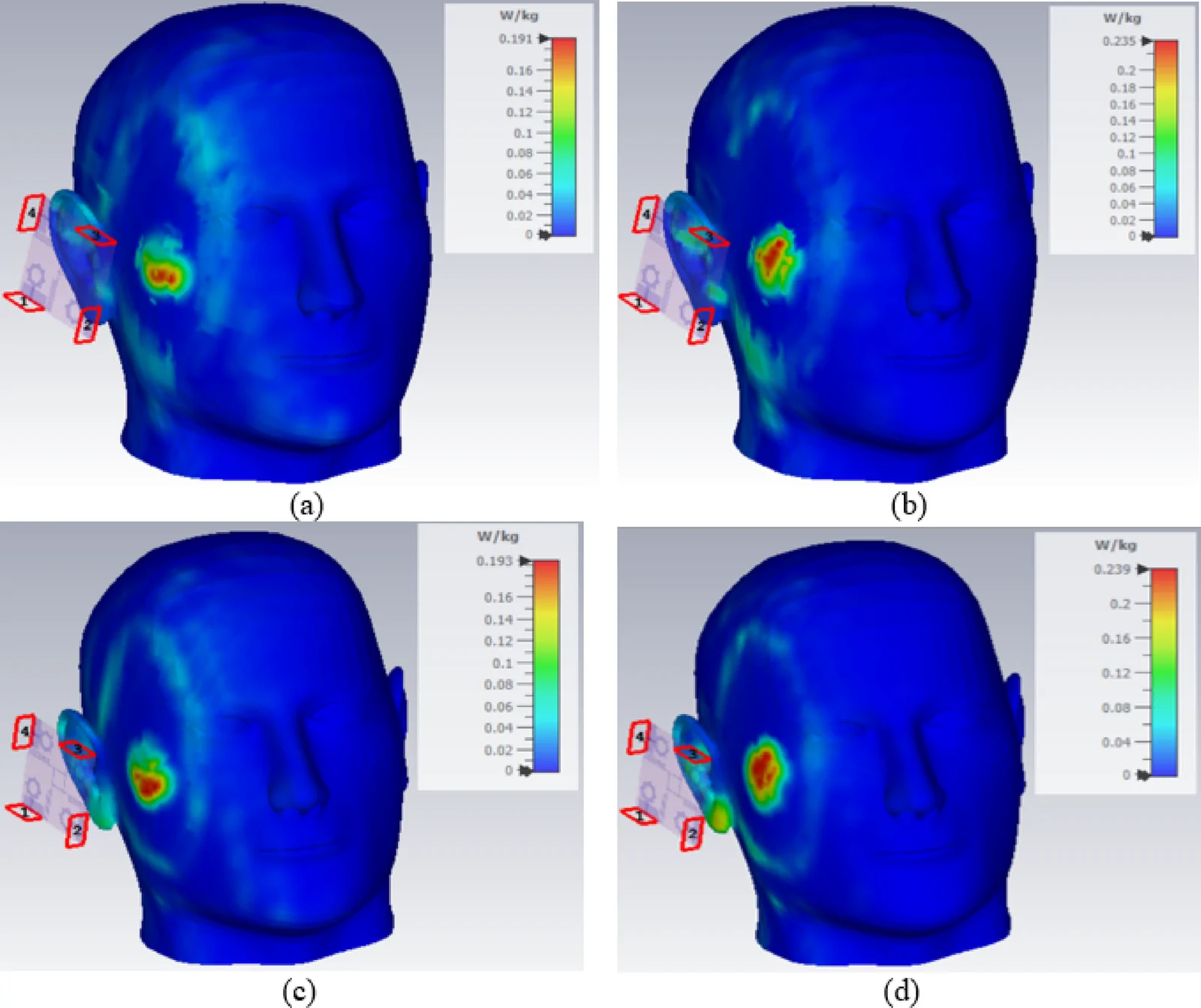
SAR analysis of the proposed MIMO antenna integrated with a human-head phantom (HHP) at (**a**) 3.50 GHz for 1 g, (**b**) 5.50 GHz for 1 g, (**c**) 3.50 GHz for 10 g, and (**d**) 5.50 GHz for 10 g.

After validating the impedance, diversity, gain, efficiency, and radiation characteristics, the safety performance of the proposed MIMO antenna is evaluated through specific absorption rate (SAR) analysis. In this analysis, the MIMO antenna is placed near the human-head phantom, and the antenna port is excited at a fixed distance of 5.00 mm from the phantom. The SAR distributions at 3.50 GHz and 5.50 GHz for 1 g and 10 g averaging masses are shown in Fig. 12, while the tissue properties and obtained SAR values are summarized in Tables 2 and 3, respectively. The SAR is calculated using Eq. (5):

5 $$\:SAR=\frac{\sigma\:{\left|E\right|}^{2}}{\rho\:}$$

where σ is the conductivity of the body tissue (S/m), E is the applied electric field (V/m), and ρ is the mass density of the body tissue (kg/m³).

The calculation of SAR depends on the conductivity of the tissue layer, the applied electric field, and the mass density of the tissue. In this study, the three-layer tissue model comprises skin, fat, and muscle, which have electromagnetic properties such as conductivity, relative permittivity, and loss tangent, as tabulated in Table 2 for two frequencies, 3.50 GHz and 5.50 GHz. Table 3 shows the SAR values of the antenna at 3.50 GHz and 5.50 GHz with an input power of 0.50 W (500 mW) for 1 g and 10 g tissue models. Figure 12(a)–(d) correspond to SAR values of 0.191 W/kg at 3.50 GHz for 1 g, 0.235 W/kg at 5.50 GHz for 1 g, 0.193 W/kg at 3.50 GHz for 10 g, and 0.239 W/kg at 5.50 GHz for 10 g, respectively. Table 3 confirms that the SAR values are within the compliance limits of less than 1.60 W/kg for 1 g tissue under FCC guidelines and less than 2.0 W/kg for 10 g tissue under ICNIRP guidelines. The use of a fixed input power, clearly defined tissue parameters, a human-head phantom model, and both 1 g and 10 g averaging masses enables fair comparison of the proposed MIMO antenna with DRA-based, handset, wearable, and other compact MIMO antenna systems reported in the literature.

**Table 2:** Electrical properties of the tissue model at selected frequencies^39^.

Tissue	Frequency (GHz)	Conductivity	Relative permittivity	Loss tangent	Density (kg/m³)
Skin	3.50	2.3081	41.473	0.28583	1109
Fat		0.15553	5.1739	0.15439	911
Muscle		2.5575	51.444	0.2553	1090
Skin	5.50	4.0478	38.994	0.33926	1109
Fat		0.27374	4.9825	0.17956	911
Muscle		4.609	48.883	0.30815	1090

**Table 3:** SAR values of the four-port MIMO antenna.

Frequency (GHz)	SAR (W/kg) (1 g)	SAR (W/kg) (10 g)	Power input (W)	Compliance (W/kg)
3.50	0.191	0.193	0.50	≤ 1.60 for FCC (1 g) ≤ 2.0 for ICNIRP (10 g)
5.50	0.235	0.239		

## State-of-the-art comparison

The proposed antenna offers several advantages over the previously reported designs listed in Table 4. Compared with many existing antennas, the proposed work provides a balanced combination of compact size, wide bandwidth, good isolation, high efficiency, low ECC, low CCL, and SAR compliance. Although some earlier works report higher gain or isolation, they are either designed for narrowband, mmWave, THz, or single-application operation. In contrast, the proposed antenna covers multiple practical wireless bands, including 5G n79, Wi-Fi 5, and Wi-Fi 6E, with a wide − 10 dB bandwidth of 3.3–7.7 GHz. The antenna maintains mutual coupling below − 17.5 dB, gain up to 5.58 dBi, and radiation efficiency of 80%–96%. Its ECC is below 1 × 10⁻⁴, and CCL is below 0.10 b/s/Hz, confirming excellent MIMO diversity performance. Moreover, unlike many compared works where SAR is not reported, the proposed design includes SAR validation for both 1 g and 10 g tissue models, confirming safe operation for user-proximate wireless devices.

**Table 4:** Comparison of the proposed work with state-of-the-art designs.

Ref.	Overall dimensions	Operating band(s)	−10 dB impedance bandwidth	Isolation (dB)	Gain (dBi)	Efficiency (%)	ECC	CCL (b/s/Hz)	SAR (W/kg)
^1^ 2022	40 × 40	3.50 5.50	3.20–5.60	> 15.0	4.90	NC	< 0.01	NC	NC
^3^ 2026	48 × 48	3.43	3.04–3.80	> 18.0	3.71	93%	< 0.010	< 0.34	NC
^4^ 2025	45.3 × 69.8	3.3,3.7,4.3, 5.8, 7.4, 8.7	3.35–3.6 3.8–4.2 4.5–5.9 6.2–7.3 7.6–8.3	> 25.0	5.5	NC	< 0.02	< 0.20	NC
^6^ 2024	56 × 56	3.45, 4.85, 6.50	2.54–3.56 4.28–4.97 5.37–8.85	> 20.0	3.20	80%	< 0.13	NC	< 2 W/kg, 10 g
^7^ 2024	41 × 41	5.90, 10.0, 15.0	1.9–20.0	> 25.5	8.0	89%	< 0.004	< 0.30	NC
^11^ 2025	52 × 30	3.52	3.18–3.81	> 26.75	2.98	91%	< 38 × 10^−5^	< 0.122	NC
^16^ 2025	44 × 44	3.50, 12.0	2.75–4.40 10.33-13.0	> 15.0	3.25	84%	< 5 × 10⁻³	< 0.35	NC
^19^ 2025	50 × 50	5.90	5.62–6.11	> 30.0	7.40	> 87.45%	< 1 × 10^−3^	< 0.40	NC
^22^ 2024	16 × 16	60.0	58.925–60.66	> 50.0	10.56	NC	< 14 × 10^−5^	< 0.007	36.8 × 10^−3^
^24^ 2022	55 × 55	5.75	5.52–6.20	> 15.0	5.20	NC	< 0.05	< 0.18	NC
^26^ 2024	20 × 19	28.0	27.5-28.35	> 19.5	5.84	NC	< 0.011	NC	NC
^27^ 2025	26 × 26	33.84, 40.28	26–40	> 25.0	9.70	NC	< 0.325	< 0.03	NC
^29^ 2025	250 μm × 259 μm	1.10 THz, 3.75 THz, 8.92 THz, 11.59 THz, 13.54 THz	(0.97–1.27) THz (3.37–3.90) THz	> 30.0	8.09	> 82%	< 1 × 10^−4^	< 0.35	NC
^41^ 2024	20 × 20	28.0	24–32	> 27.0	8.24	94%	< 6 × 10^−4^	NC	NC
^49^ 2026	104 × 64	5.90	5.12–6.72	> 18.0	4.90	NC	< 0.002	< 0.30	NC
**Proposed**	60 × 60	5G NR n79 Wi-Fi 5 Wi-Fi 6E	3.3–7.7	> 17.5	**5.58**	80%–96%	**< 1 × 10**^**−4**^	**< 0.10**	0.191 (1 g), 0.193 (10 g) at 3.50 GHz 0.235 (1 g), 0.239 (10 g) at 5.50 GHz

## Conclusions

This article presents a compact modified octagonal ring-shaped four-port MIMO antenna with a connected-ground structure for 5G sub-6 GHz n79, Wi-Fi 5, and Wi-Fi 6E wireless applications. The proposed antenna is designed on a low-cost FR4 substrate with an overall size of 60 × 60 mm², making it suitable for compact wireless devices. The orthogonal placement of the four radiating elements improves spatial and polarization diversity, while the connected-ground structure with additional strips and slits enhances impedance matching and reduces mutual coupling among the antenna ports. The antenna provides a wide operating bandwidth from approximately 3.3 GHz to 7.7 GHz, successfully covering the targeted wireless bands. The measured and simulated S-parameters show good agreement, with mutual coupling maintained below − 17.5 dB across the operating band. The radiation characteristics of the antenna are stable over the desired frequency range, with peak realized gain ranging from about 4.3 dBi to 5.58 dBi and radiation efficiency varying between 80 and 96%. The 2D and 3D radiation patterns confirm nearly omnidirectional and stable radiation behavior, which is suitable for practical MIMO communication systems. The diversity parameters, including ECC, DG, TARC, MEG, and CCL, remain within acceptable limits, confirming the suitability of the proposed structure for high-performance MIMO operation. In addition, SAR analysis using a human-head phantom model shows that the antenna satisfies FCC and ICNIRP safety standards for both 1 g and 10 g tissue models. Overall, the proposed antenna offers a good combination of compactness, wide bandwidth, high isolation, stable gain, good efficiency, reliable diversity performance, and SAR compliance, making it an appropriate candidate for modern 5G and WLAN communication devices.

## Data Availability

The datasets used and/or analyzed during the current study are available from the corresponding author on reasonable request.
